# Scrub typhus complicated by cholecystitis: a case report

**DOI:** 10.3389/fsurg.2026.1753759

**Published:** 2026-04-14

**Authors:** Yuliang Wang, Shaojuan Lai, Zhiqiang Wang, Zhixin Cui, Jianhua Liu

**Affiliations:** Department of Critical Care Medicine, Guangzhou Panyu District Maternaland Child Health Hospital (Affiliated Hospital Group of Guangdong Medical University Panyu HeXian Memorial Hospital), Guangzhou, Guangdong, China

**Keywords:** cholecystitis, diagnostic, eschar, scrub typhus, tNGS

## Abstract

**Objective:**

Scrub typhus complicated by acute cholecystitis is rare. Scrub typhus lacks specific clinical manifestations in its early stage, especially in patients without eschars, requiring comprehensive diagnosis based on epidemiological history, clinical presentation, and laboratory results. This case report describes a patient with scrub typhus complicated by acute cholecystitis, ultimately confirmed by tNGS, reminding surgeons to emphasize physical examination and avoid diagnostic anchoring.

**Methods:**

We report a case of a 74-year-old female who presented with right upper quadrant pain and fever. Laboratory tests showed elevated white blood cell count and increased neutrophil percentage, while CT suggested chronic cholecystitis. The patient received anti-infective therapy with cefoperazone sodium and sulbactam sodium, as well as gallbladder puncture and drainage. However, her condition continued to deteriorate, with the development of multiple organ dysfunction syndrome (MODS) and disseminated intravascular coagulation (DIC). She resided in a rural area of Guangzhou, China, but had not engaged in agricultural work. After transfer to the intensive care unit, an eschar was discovered on her lower back. The diagnosis was confirmed by targeted next-generation sequencing (tNGS) of blood samples. Active interventions including doxycycline anti-infective therapy, mechanical ventilation, blood purification, and blood transfusion were subsequently administered.

**Results:**

The patient's condition continued to deteriorate. During her ICU stay, she experienced one episode of cardiac arrest. Although spontaneous circulation was restored through aggressive resuscitation, the shock state remained refractory to correction. Given the poor prognosis and the unbearable burden of continued treatment, rehabilitation, and nursing care, the family ultimately chose to withdraw life-sustaining therapy.

**Conclusions:**

This case illustrates a patient with scrub typhus whose initial clinical presentation was an attack of cholecystitis. Delayed diagnosis led to rapid progression and deterioration of the patient's condition, ultimately resulting in death. This case reminds us that surgeons need to enhance their awareness of local epidemic diseases during outbreak seasons, emphasize physical examination, and promptly employ advanced diagnostic methods such as tNGS for difficult cases, thereby reducing the risks of misdiagnosis and missed diagnosis.

## Introduction

Scrub typhus is an acute zoonotic infectious disease caused by *Orientia tsutsugamushi infection,* with universal susceptibility in the general population. The scrub typhus pathogen lacks lipopolysaccharide and is an obligate intracellular bacterium with unique biological characteristics. This bacterium can spread systemically, damaging vascular endothelial cells, promoting the production of pro-inflammatory cytokines, and inducing inflammatory responses that lead to organ function impairment (1). Its clinical manifestations include fever, systemic toxic symptoms, characteristic eschar and ulceration, lymphadenopathy, hepatosplenomegaly, and rash. Laboratory diagnostic methods include the Weil-Felix test, indirect immunofluorescence assay, blood culture, and quantitative polymerase chain reaction (qPCR) (2). Second-generation sequencing technology, particularly tNGS, has emerged as a novel tool for the detection of scrub typhus (3). A diagnosis of scrub typhus can be confirmed in clinically suspected cases when at least one laboratory test yields a positive result. In untreated individuals, the infection may lead to widespread organ damage, presenting as interstitial pneumonia, hepatic injury, myocarditis, meningitis, and other complications (4). Therefore, in patients lacking eschars, the clinical and laboratory features of scrub typhus closely resemble those of many other infectious diseases, often resulting in delayed diagnosis and poor outcomes (5). In the etiological diagnosis of fever of unknown origin and suspected coinfections, tNGS offers distinct advantages: it is faster than conventional microbial culture, and unlike serological assays or PCR, it enables unbiased, high-throughput sequencing of nucleic acids from clinical specimens without requiring prior suspicion of specific pathogens. Through a case of scrub typhus ultimately diagnosed using tNGS, this paper reviews the diagnostic challenges encountered and highlights the clinical value of tNGS in managing febrile illnesses, emphasizing the need for surgeons to expand their differential diagnostic reasoning.

## Detailed case description

### Initial presentation

**Figure 1:**
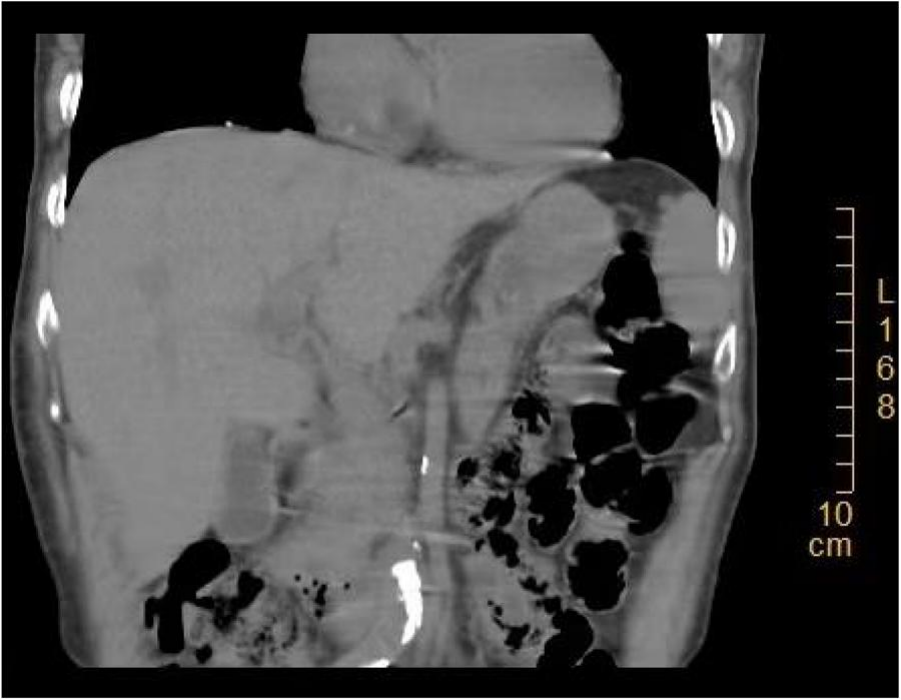
The upper abdominal CT scan revealed a postprandial gallbladder with a slightly thickened and coarse gallbladder wall.

On August 11, 2025, a 74-year-old woman presented to the emergency department with a one-day history of right upper quadrant pain and fever. She denied nausea, vomiting, dyspnea, diarrhea, or obstipation. Her medical history included hypertension, which was well-controlled with regular medication (Amlodipine Tablets). She lived in a rural area but denied specific exposure risks related to agricultural work. There was no history of liver disease, cholelithiasis, or choledocholithiasis. She also denied smoking, alcohol abuse, or any family history of malignancy. An abdominal CT scan revealed slight thickening and irregularity of the gallbladder wall, along with mild intrahepatic biliary duct dilation Figure 1. She was admitted to the Hepatobiliary Surgery department with a diagnosis of acute exacerbation of chronic cholecystitis.

### Hospital course

(DAY0) On admission, her vital signs were: temperature 37.5 °C, pulse 116 beats/min, respiratory rate 20 breaths/min, blood pressure 83/57 mmHg, and oxygen saturation 97%. Physical examination showed no jaundice or skin rash. The abdomen was flat, without visible venous collaterals, peristaltic waves, or old surgical scars. Abdominal muscles were soft, with tenderness in the right upper quadrant, no rebound tenderness, and a positive Murphy's sign. The liver and spleen were not palpably enlarged, bowel sounds were normal, and shifting dullness was negative. Lungs auscultation was clear without rales. The remainder of the physical exam was within normal limits. Relevant laboratory tests were performed on admission (Table 1).

**Table 1:** Laboratory parameters during hospitalization.

Parameter (Units)	DAY0	DAY2	DAY3	Normal Range
WBC (x10^9^/L)	10.38	11.55	6.44	3.5–9.5
Neutrophils (%)	91.5	91.0	32.5	40–75
Lymphocytes (%)	4.5	5	61.3	20–50
Eosinophils (%)	0.1	0	0.1	0.4–8.0
Haemoglobin (g/L)	121	112	35	115–150
Plateles (x10^9^/L)	124	45	21	125–350
PCT (ng/mL)	NA	7.24	6.15	<0.05
CRP (mg/L)	72.8	94.09	72.06	0–6
BUN (mmol/L)	10.3	16.8	20.9	2.76–8.07
Creatinine(μmol/L)	115	227	282	44–80
AST/ALT (U/L)	105/69	157/73	188/56	
LDH/CK (U/L)	699/331	891/135	743/160	
CK-MB (U/L)	35	17	19	0–25
Lac (mmol/L)	2.45	3.15	11.95	0.5–2.44
PT (sec)	11.5	16.2	30.6	9.7–12.6
APTT (sec)	30.7	62.7	120	25.0–31.3
Fibrinogen (g/L)	2.83	1.23	0.79	1.8–3.5
D-Dimer	NA	10.68	NA	0–0.55
BNP (pg/mL)	29.68	56.51	85.89	<100

DAY1 First visit, WBC, white blood cell; Hb, haemoglobin; PCT, procalcitonin; CRP, C-reactive protein; BUN, blood urea nitrogen; AST, aspartate aminotransferase; ALT, alanine transaminase alaninetransaminase; LDH, lactate dehydrogenase; CK, creatine kinase; CK-MB, creatine kinase isoenzyme; Lac, lactic acid; PT, prothrombin time; APTT, activated partial thromboplastin time; BNP, B-type natriuretic peptide; NA, not applicable or not available because the test were not ordered.

**Figure 2:**
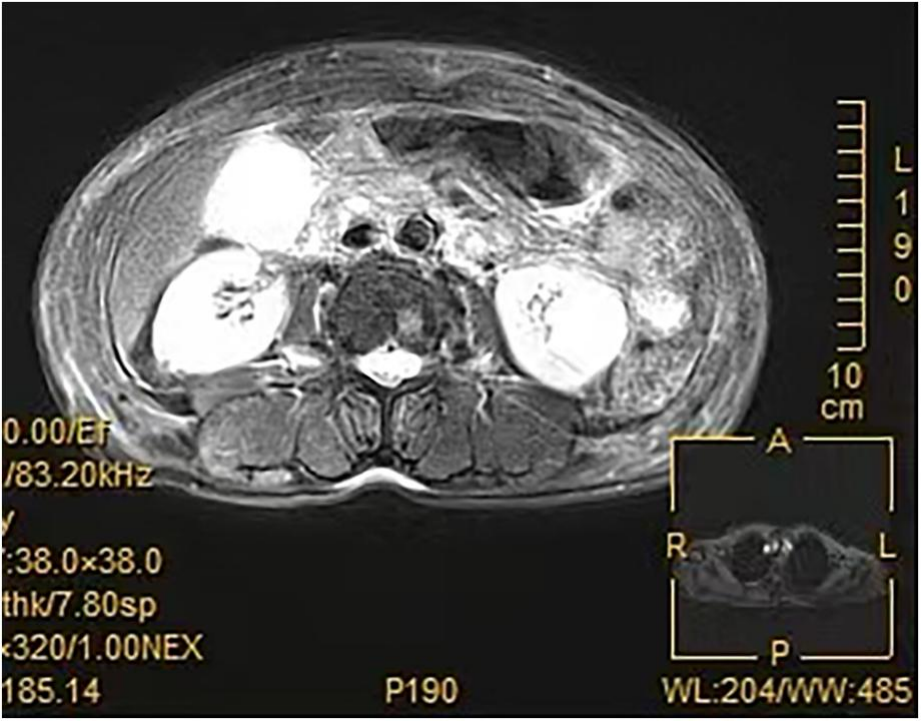
MRCP of the gallbladder revealed gallbladder distension, thickening of the gallbladder wall, and a small amount of fluid in the gallbladder fossa.

Due to the acute onset of right epigastric pain, Murphy's sign positive, fever and leukocytosis, combined with CT results showing acute attack of chronic cholecystitis, the surgeon initiated intravenous cefoperazone-sulbactam after completing blood culture testing. Because of the patient's low blood pressure, fluid resuscitation and norepinephrine tartrate were carried out at the same time to raise blood pressure.

(DAY1) The patient remained febrile and vasopressor-dependent. She developed oliguria and edema of the face and limbs. An abdominal MRI supported the diagnosis of acute exacerbation of chronic cholecystitis and noted Glisson's sheath edema Figure 2. An infectious disease consultation was sought. Physical examination revealed no rash. Considering the high local prevalence of dengue fever, hemorrhagic fever, and Chikungunya fever during that season, regional dietary habits (consumption of raw fish), and the presentation of acute febrile illness with multi-organ dysfunction (liver impairment, elevated cardiac enzymes, thrombocytopenia, and elevated serum creatinine), further tests were ordered. Serology for hemorrhagic fever was negative for both IgG and IgM, Clonorchis sinensis antibody was negative, Chikungunya virus nucleic acid test was negative, and dengue virus NS1 antigen test was negative.

(DAY2) The patient still has bdominal pain, fever, and vasopressor requirement persisted. Diuretic administration did not significantly improve urine output, and facial and limb edema worsened. Repeat labs showed increased WBC and PCT, with marked deterioration in coagulation profile and renal function (Table 1). The surgeons performed Percutaneous transhepatic gallbladder drainage (PTGD). That evening, the patient developed respiratory distress and was transferred to the ICU for further management.

### ICU management

**Figure 3:**
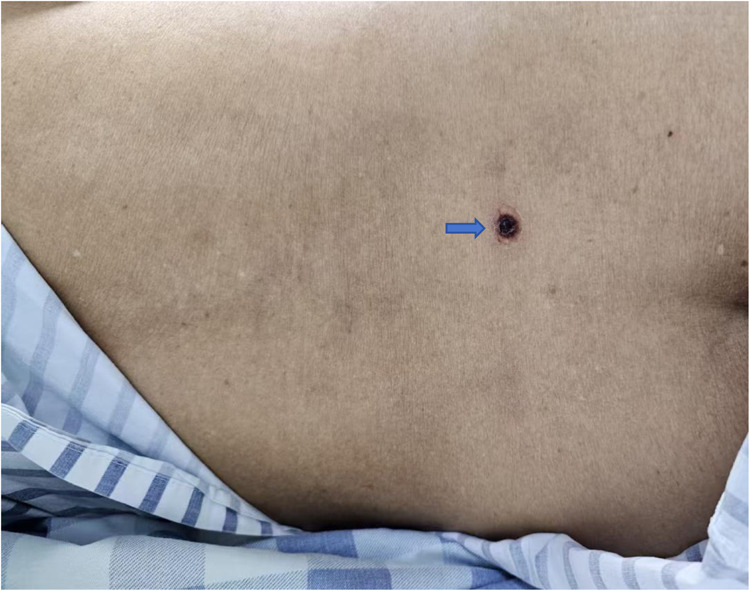
On the second day of hospitalization, a crater-shaped eschar were found on the patient's Lumbar back appearance with central necrosis.

(DAY2, At night) In the ICU, physical examination revealed a crater-like eschar on the skin of the lower back (Figure 3), raising strong suspicion of scrub typhus complicating acute cholecystitis. Targeted antimicrobial therapy with intravenous imipenem-cilastatin and oral doxycycline was initiated. The patient was intubated for mechanical ventilation and started on continuous renal replacement therapy (CRRT) and ongoing fluid resuscitation. Due to hemorrhagic output from the cholecystostomy drain and documented coagulopathy, the patient received blood product transfusions. At the same time, blood was collected for tNGS detection.

(DAY3) The tNGS results of the blood sample revealed *Orientia tsutsugamushi* (6 × 10⁵ copies/mL, 150,098 reads) and Epstein–Barr virus (1 × 10⁴ copies/mL, 3,412 reads). The lowest limit of this technology for detecting pathogenic microorganisms is 50 copies/mL. In the case of EB virus infection, the common symptoms include fever and sore throat, and the blood routine can show the increase of lymphocytes. The patient has no symptoms of upper respiratory infection and no obvious increase of lymphocytes, so it is not treated for the time being. In particular, disseminated intravascular coagulation (DIC) was not controlled, and bloody fluid was drained via the percutaneous transhepatic gallbladder puncture tube. Follow-up complete blood count and coagulation function tests revealed a significant decrease in hemoglobin, a marked reduction in platelets. Septic shock complicated with hemorrhagic shock was considered. Although active fluid resuscitation, transfusion of suspended red blood cells, fresh frozen plasma and cold precipitation were carried out in time, high-dose norepinephrine bitartrate was still required to maintain blood pressure, and the bleeding tendency was not improved. The patient experienced a cardiac arrest. Although the vital signs were restored after timely cardiopulmonary resuscitation, the organ dysfunction of the patient continued to progress.

### Outcome

(DAY3) After thorough communication with the patient's family regarding the critical condition, they were informed that the required comprehensive treatment and intensive care would involve considerable expense. Even with continued therapy, there is a risk of death at any time due to unstable multi-organ function. Considering the poor prognosis and the unbearable burden of ongoing treatment, rehabilitation, and nursing care, the family finally chose to stop treatment.

Two sets of blood cultures drawn during hospitalization showed no growth after five days. The progress of patients during hospitalization was summarized in tabular form Table 2.

**Table 2:** Patient's visit timeline.

Timeline	Key clinical events	Diagnostic	Interventions
Initial Presentation	The patient presented to the emergency department with right upper quadrant pain and fever for one day. An upper abdominal CT scan revealed a slightly thickened and coarse gallbladder wall, along with mild intrahepatic bile duct dilatation	Acute exacerbation of chronic cholecystitis	The patient was admitted to the Hepatobiliary Surgery Department for inpatient treatment
DAY0	The patient presented with hypotension (83/57 mmHg), tachycardia (116 bpm), fever, and a positive Murphy's sign	Acute exacerbation of chronic cholecystitis	Relevant blood tests were performed. The patient was administered intravenous cefoperazone-sulbactam sodium, while also initiating fluid resuscitation and norepinephrine bitartrate to maintain blood pressure.
DAY1	Physical examination revealed reduced urine output and edema involving the face and limbs	Acute exacerbation of chronic cholecystitis	Norepinephrine bitartrate was administered to maintain blood pressure. Abdominal MRI suggested acute exacerbation of chronic cholecystitis and edema of Glisson's capsule.
DAY2	The patient continued to experience abdominal pain and fever and required vasopressors to maintain blood pressure. Follow-up tests showed continued elevation of WBC and PCT	Acute exacerbation of chronic cholecystitis	PTGD
DAY2, At night	The patient was transferred to the ICU due to dyspnea; a crater-like eschar was found on the skin of the lower back	Scrub typhus; Acute exacerbation of chronic cholecystitis; Septic shock; MOF	Doxycycline was added for anti-infective therapy, along with blood pressure maintenance, fluid resuscitation, and blood purification. Blood samples were sent for tNGS to identify the pathogen
DAY3	WBC and PCT showed improvement, but MOF remained unimproved	Scrub typhus; Acute exacerbation of chronic cholecystitis; Septic shock; MOF	The patient underwent one successful cardiopulmonary resuscitation (CPR). The family finally chose to stop treatment

## Discussion

In China, scrub typhus was first reported in Guangzhou in 1948. In recent years, the incidence of scrub typhus in various regions of China has been increasing year by year, and it can occur all the year round in Guangdong Province, with the highest incidence in summer (6). In recent years, cases of scrub typhus have been reported in Africa, France, the Middle East, and South America, indicating a gradual expansion of its endemic regions. This expanding epidemiological pattern underscores the need for increased awareness and further research on scrub typhus (7). During periods of active transmission, clinicians should maintain a high index of suspicion for this disease in patients presenting with fever, eschar or ulceration, lymphadenopathy, and hepatosplenomegaly—especially if there is a history of exposure to endemic areas (8). However, due to the nonspecific nature of clinical manifestations, diagnosis cannot rely solely on symptoms and must be confirmed through laboratory testing. In this case, an eschar was identified only after transfer to the intensive care unit, and the diagnosis was ultimately confirmed by tNGS. Despite initiation of targeted anti-infective therapy, the patient's condition continued to deteriorate rapidly, progressing to septic shock, MODS, and DIC, ultimately resulting in death. This outcome highlights the high fatality rate associated with scrub typhus when clinical presentation is atypical and diagnosis is delayed.

This patient initially presented with right upper quadrant pain, fever, and a positive Murphy's sign. The pre-admission CT scan demonstrated gallbladder wall thickening, and laboratory tests revealed leukocytosis. An MRI on the second hospital day confirmed acute exacerbation of chronic cholecystitis. Despite initiation of antimicrobial therapy with cefoperazone-sulbactam, the patient's condition failed to improve. She subsequently developed hepatic dysfunction, acute kidney injury, and DIC, ultimately progressing to multiple organ failure. Indeed, although scrub typhus complicated by acute cholecystitis (including both calculous and acalculous forms) is uncommon, it has been well documented in several case reports. Reported cases typically respond well to targeted antibiotics, with no fatalities (9–11). Currently, the mechanism of tsutsugamushi disease complicated with acute cholecystitis remains unclear. Studies suggest that it may be associated with systemic vasculitis and vascular endothelial injury of the gallbladder wall. Like other complications, vascular endothelial injury promotes the production of pro-inflammatory cytokines (e.g., tumor necrosis factor α), which leads to the aggregation of WBC, particularly neutrophils.These neutrophils secrete chemokines and effector proteins that induce an inflammatory response, rather than direct bacterial infection (1, 12). This case also consistent with the characteristics of this pathogenesis. Early empirical use of cefoperazone-sulbactam sodium was ineffective, WBC persisted, and MRCP showed inflammatory exudation in the gallbladder fossa. Although pathognomonic for scrub typhus, the eschar is frequently not the initial presenting symptom due to its painless and non-pruritic characteristics. The diagnostic process is further complicated by the non-specific and overlapping manifestations of common co-infections, often resulting in delayed identification of the disease. Studies have reported a median interval of 5 days (interquartile range: 2–9 days) from symptom onset to definitive diagnosis of scrub typhus, and such diagnostic delays may lead to the administration of inappropriate antibiotics during the early infection phase, potentially exacerbating disease severity (13, 14). If the physician promptly establishes a definitive diagnosis and initiates specific treatment measures, the mortality rate can be significantly reduced (1.8%). Without timely and appropriate treatment, severe infection patients with hypoperfusion are prone to multiple organ failure and even death. It has been reported that the mortality rate of severe cases is 30%–70% (8, 15). In this case, targeted anti-rickettsial therapy was initiated on day 3 of illness (hospital day 2), yet the patient failed to respond, with progressive MODS and refractory DIC. Cases of treatment failure in scrub typhus complicated by acute cholecystitis have rarely been reported. The treatment failure in this case was attributed to refractory disseminated intravascular coagulation (DIC). After the patient was transferred to the ICU, it was noted that fresh blood was continuously draining from the percutaneous transhepatic gallbladder drainage tube. Follow-up tests revealed a significant decrease in hemoglobin and platelet levels, along with coagulation dysfunction. Although timely transfusions of packed red blood cells, fresh frozen plasma, and cryoprecipitate were administered, the bleeding symptoms did not improve. Previous reports have documented cases of scrub typhus complicated by splenic rupture, ultimately resulting in death due to severe disseminated intravascular coagulation (DIC). In those cases, autopsy findings revealed systemic vasculitis and perivasculitis (15). The occurrence of DIC in scrub typhus may be associated with multiple mechanisms, including: Orientia tsutsugamushi releases an endotoxin-like toxin that damages vascular endothelial cells, leading to microvascular proliferation and microthrombus formation. In addition, the host's severe immune response to O. tsutsugamushi amplifies the inflammatory reaction, contributing to the development of DIC.

The eschar, a hallmark sign of scrub typhus, arises from necrosis at the site of a mite bite and is histopathologically characterized by superficial crusting and perivascular inflammatory cell infiltration within the dermis (5). Eschars are most commonly observed in the groin, perineal/perianal region, and buttocks; they are also frequently seen on the breasts and inframammary areas in females. Additionally, eschars may be found on other sites including the neck, abdomen, chest, and various skin folds (16). This distribution correlates with the ecological preferences of larvae for regions abundant in apocrine glands. The precise timing of eschar appearance remains poorly defined; however, it may precede clinical symptoms by 2–3 days. Not all patients exhibit a classic eschar, with reported prevalence rates varying significantly from 28.5% to 93% (16, 17). The detection of an eschar has been noted to correlate with skin pigmentation; studies indicate that identification is more challenging in individuals with darker skin tones. Furthermore, early-stage lesions or those that have healed or been altered due to prior antibiotic exposure are particularly prone to being overlooked (18). In our case study, an eschar was identified on the lower back during examination in the ICU setting. A review of surgical ward notes and nursing records revealed no previous documentation regarding any rash. Upon communicating with the hepatobiliary surgeon to verify the patient's skin rash, the attending physician acknowledged that a comprehensive skin examination had not been performed during hospitalization. Therefore, the delayed identification may have been due to the inconspicuous location of the eschar and the lack of a thorough physical examination. Conducting a detailed full-body skin examination in suspected cases is a critical step in preventing missed diagnoses.

Diagnosis of scrub typhus relies heavily on laboratory testing. Conventional culture methods have a limited ability to detect fastidious pathogens and are often time-consuming. Serological methods like indirect fluorescence have low sensitivity, and since antibody levels are dependent on the timing of infection, they pose a risk of false-negative results (17). The indirect immunofluorescence assay (IFA) is the gold standard for diagnosing scrub typhus; however, its application is limited due to high cost and technical complexity (19). Currently, the most common diagnostic method for scrub typhus is qPCR. However, its application is limited to cases with a strong clinical suspicion of Orientia tsutsugamushi infection. Since qPCR primarily relies on specific primers to exponentially amplify known, predetermined DNA sequences, it can only target specific pathogens. This mechanism confers an inherent limitation in detecting co-infections. In this case, the rapid detection of Orientia tsutsugamushi via blood tNGS, with a high sequence count of 150,098, provided a decisive basis for clinical decision-making. Compared to traditional serology, tNGS offers advantages of being unbiased, high-throughput, rapid, and relatively cost-effective, making it particularly suitable for fever of unknown origin, unusual pathogens, critical sepsis, and polymicrobial infections (2, 20, 21). The 2021 international guidelines for management of sepsis and septic shock recommend completing etiological screening before administering antibiotics. Currently, the optimal timing for tNGS testing in sepsis has not been clearly established. However, a Chinese Expert consensus on the application and practice of targeted next-generation sequencing in infectious diseases mentions that, as a supplementary method to traditional etiological screening, tNGS can obtain results faster and has a higher positive rate (22, 23).

However, limitations of tNGS must be acknowledged. This technology uses ultra-multiplexed PCR coupled with NGS to identify pathogens in a sample, covering hundreds of common clinical microbes including bacteria, fungi, and viruses. This pathogen detection method has a defined lower limit of detection. Samples with microbial concentrations below this threshold may yield false-negative results, particularly in early infection stages or when specimens are collected from suboptimal sites. The technique relies on existing genomic databases for probe design; novel or highly divergent pathogens might be missed. Contamination during non-sterile collection can lead to detection of environmental organisms. Thus, results require careful interpretation in the clinical context.

## Conclusions

In endemic areas, patients presenting with fever and multi-organ involvement during summer and autumn should undergo daily, thorough skin examinations. Even in the absence of a detected eschar, scrub typhus should remain a strong consideration to avoid diagnostic anchoring. For febrile patients failing initial therapy, those with MODS, or suspected co-infections, early tNGS testing is recommended, potentially avoiding delays associated with waiting for serological results. Furthermore, enhancing recognition and training regarding scrub typhus among primary care physicians and surgeons is of significant practical importance.

## Data Availability

The original contributions presented in the study are included in the article/Supplementary Material, further inquiries can be directed to the corresponding author.
